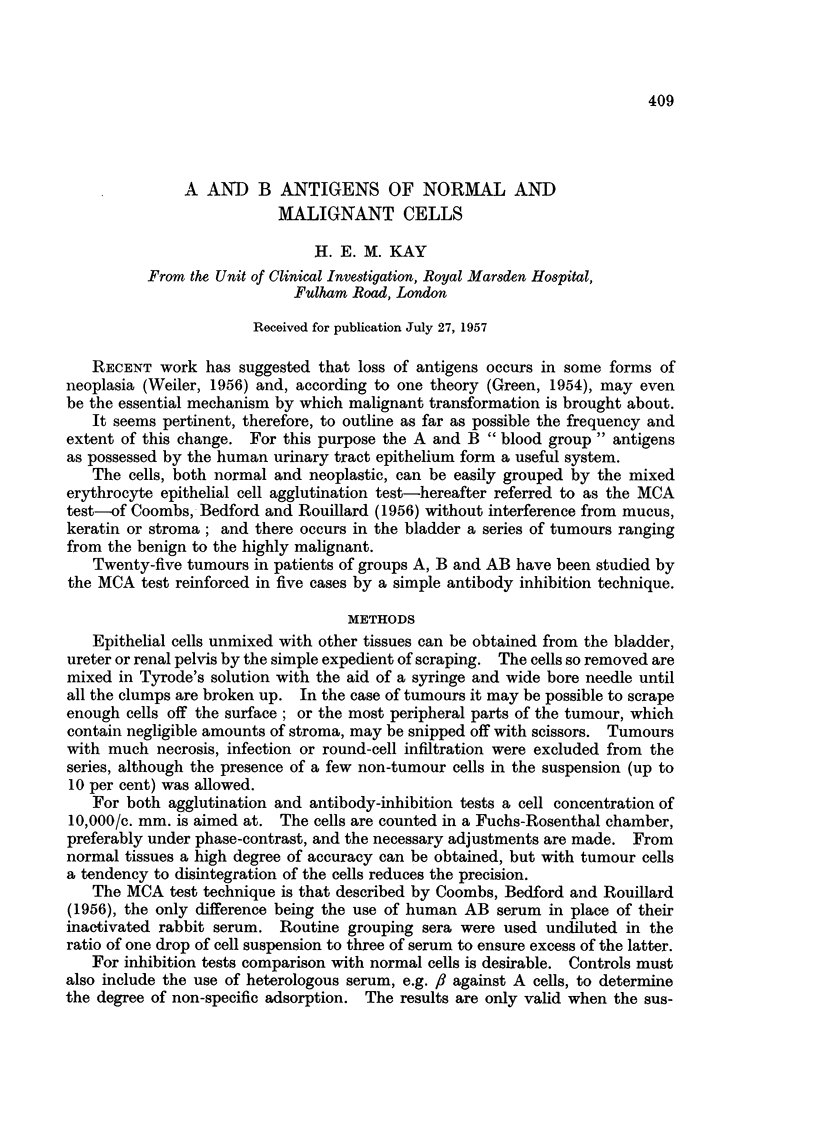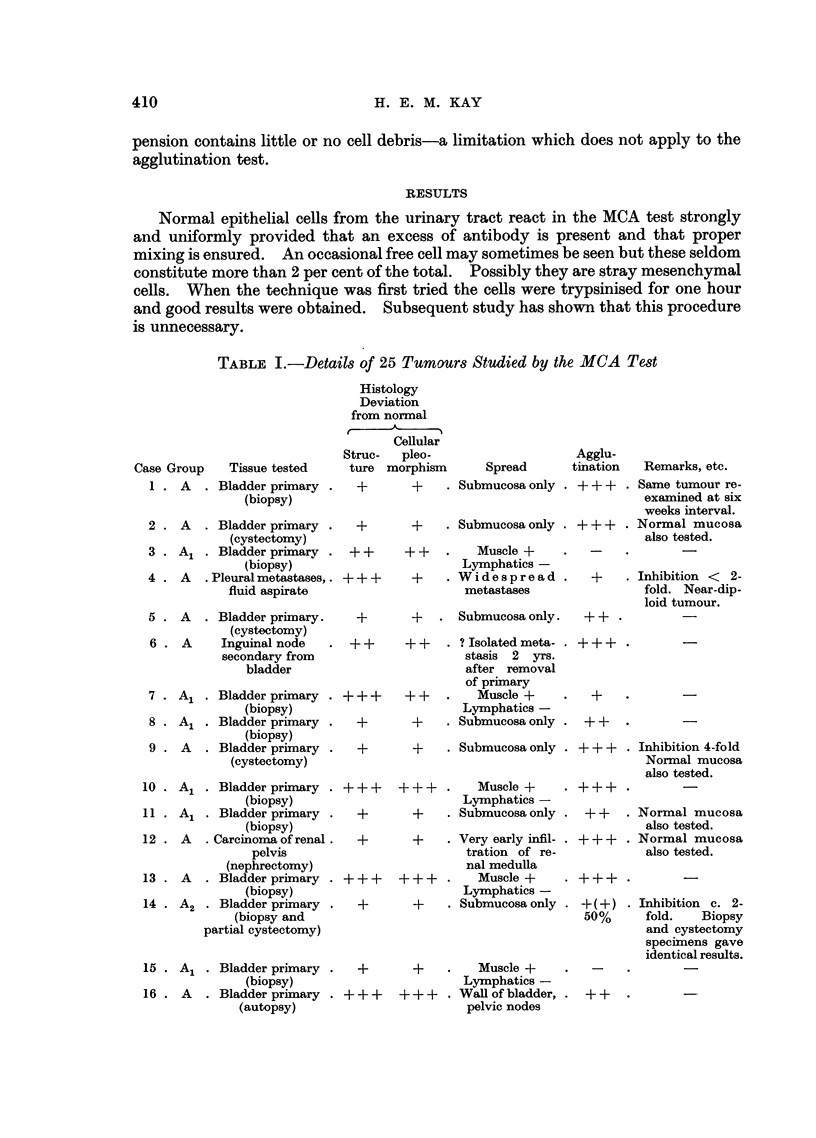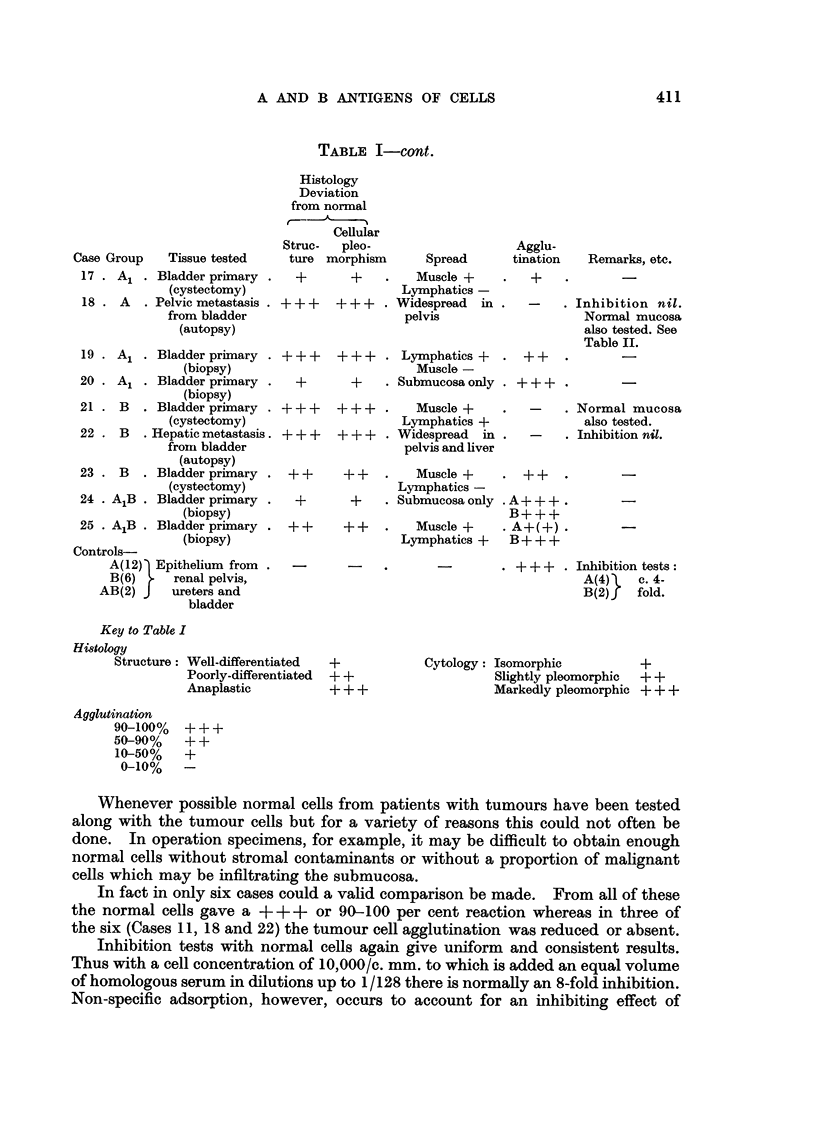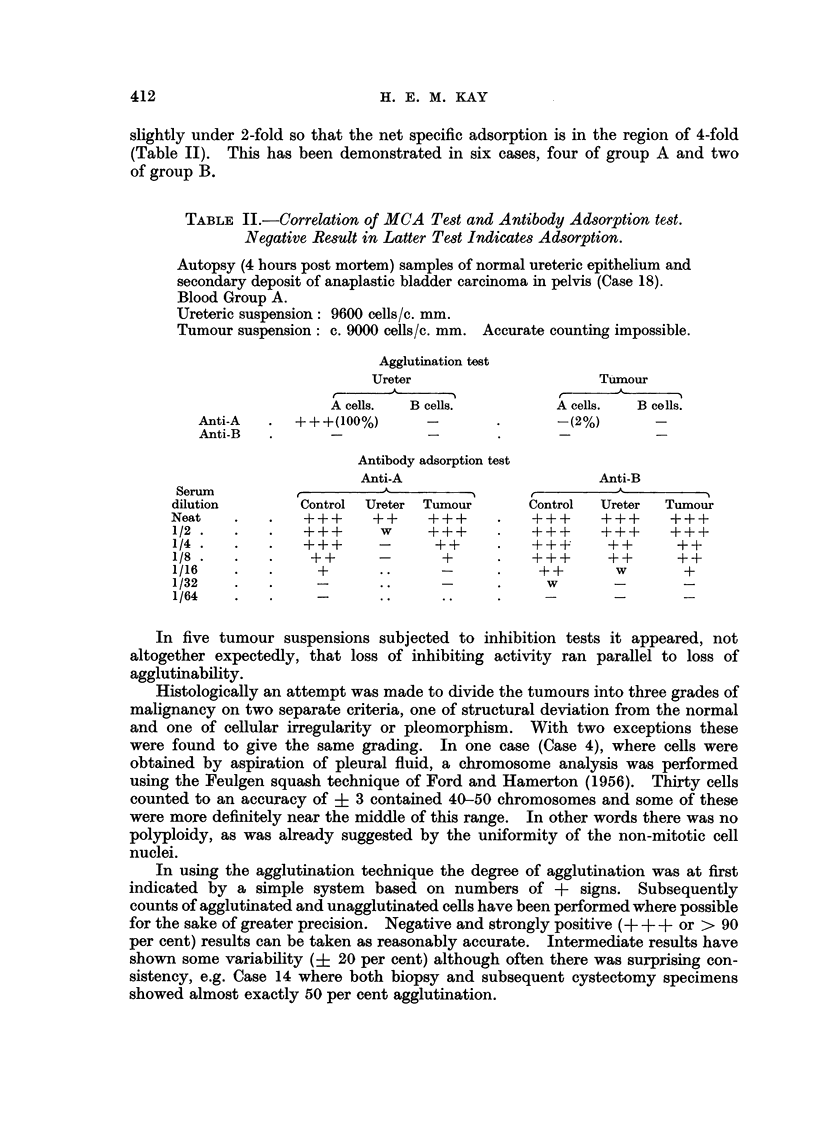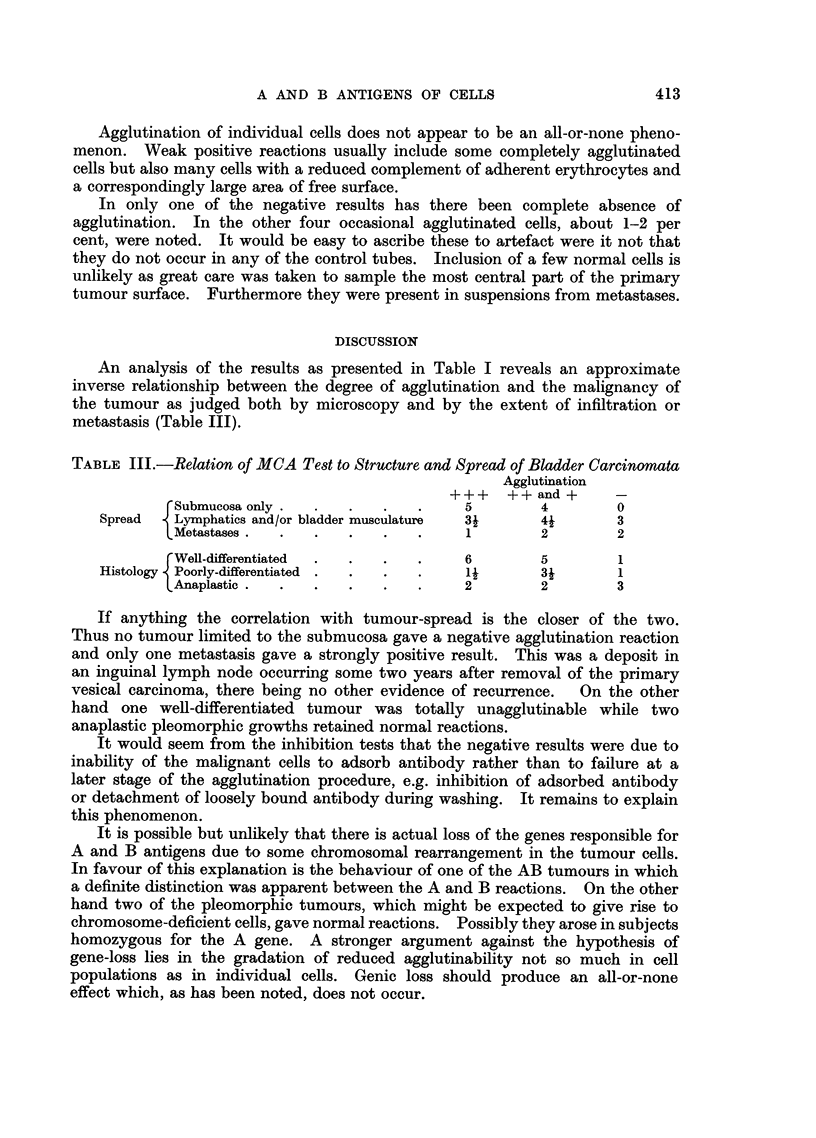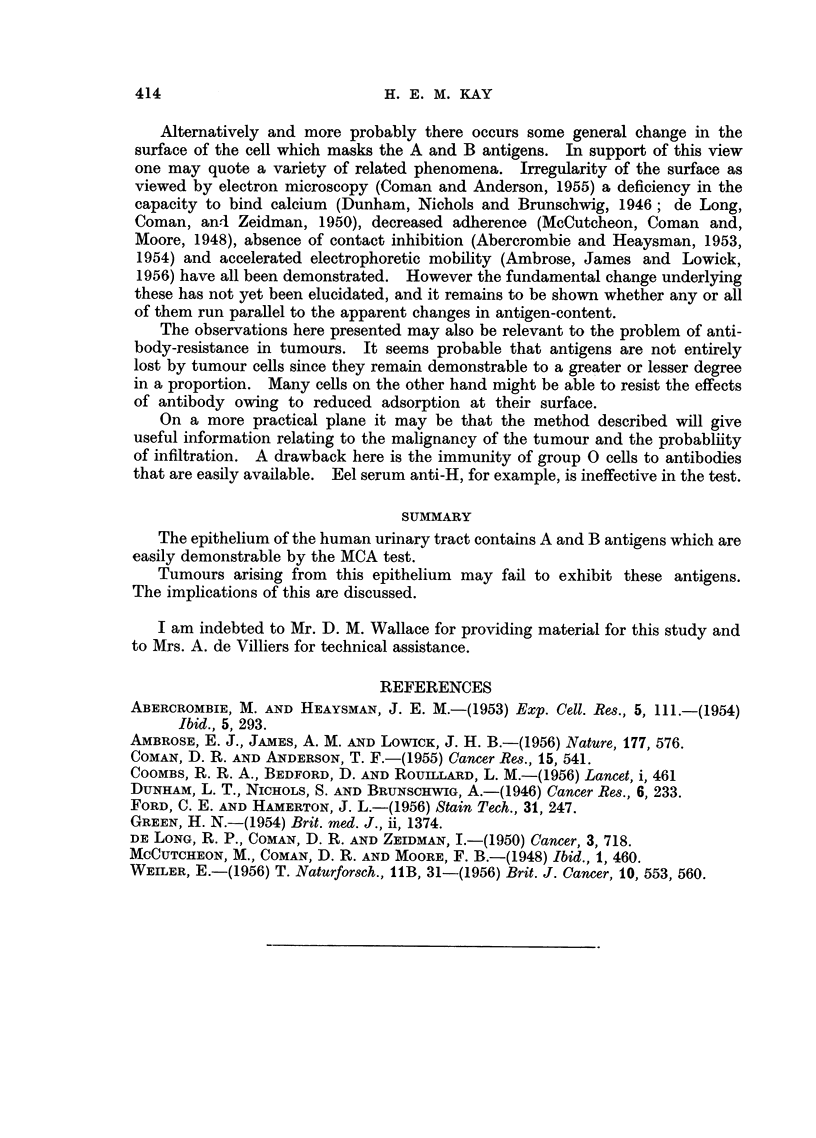# A and B Antigens of Normal and Malignant Cells

**DOI:** 10.1038/bjc.1957.49

**Published:** 1957-09

**Authors:** H. E. M. Kay


### A AND B ANTIGENS OF NORMAL AND MALIGNANT CELLS

H. E. M. KAY

*From the Unit of Clinical Investigation, Royal Marsden Hospital, Fulham Road, London*

Received for publication July 27, 1957

RECENT work has suggested that loss of antigens occurs in some forms of neoplasia (Weiler, 1956) and, according to one theory (Green, 1954), may even be the essential mechanism by which malignant transformation is brought about. It seems pertinent, therefore, to outline as far as possible the frequency and extent of this change. For this purpose the A and B " blood group " antigens as possessed by the human urinary tract epithelium form a useful system.

The cells, both normal and neoplastic, can be easily grouped by the mixed erythrocyte epithelial cell agglutination test—hereafter referred to as the MCA test—of Coombs, Bedford and Rouillard (1956) without interference from mucus, keratin or stroma ; and there occurs in the bladder a series of tumours ranging from the benign to the highly malignant.

Twenty-five tumours in patients of groups A, B and AB have been studied by the MCA test reinforced in five cases by a simple antibody inhibition technique.

### METHODS

Epithelial cells unmixed with other tissues can be obtained from the bladder, ureter or renal pelvis by the simple expedient of scraping. The cells so removed are mixed in Tyrode's solution with the aid of a syringe and wide bore needle until all the clumps are broken up. In the case of tumours it may be possible to scrape enough cells off the surface ; or the most peripheral parts of the tumour, which contain negligible amounts of stroma, may be snipped off with scissors. Tumours with much necrosis, infection or round-cell infiltration were excluded from the series, although the presence of a few non-tumour cells in the suspension (up to 10 per cent) was allowed.

For both agglutination and antibody-inhibition tests a cell concentration of 10,000/c. mm. is aimed at. The cells are counted in a Fuchs-Rosenthal chamber, preferably under phase-contrast, and the necessary adjustments are made. From normal tissues a high degree of accuracy can be obtained, but with tumour cells a tendency to disintegration of the cells reduces the precision.

The MCA test technique is that described by Coombs, Bedford and Rouillard (1956), the only difference being the use of human AB serum in place of their inactivated rabbit serum. Routine grouping sera were used undiluted in the ratio of one drop of cell suspension to three of serum to ensure excess of the latter.

For inhibition tests comparison with normal cells is desirable. Controls must also include the use of heterologous serum, e.g. $\beta$ against A cells, to determine the degree of non-specific adsorption. The results are only valid when the suspension contains little or no cell debris—a limitation which does not apply to the agglutination test.

### RESULTS

Normal epithelial cells from the urinary tract react in the MCA test strongly and uniformly provided that an excess of antibody is present and that proper mixing is ensured. An occasional free cell may sometimes be seen but these seldom constitute more than 2 per cent of the total. Possibly they are stray mesenchymal cells. When the technique was first tried the cells were trypsinised for one hour and good results were obtained. Subsequent study has shown that this procedure is unnecessary.

TABLE I.—*Details of 25 Tumours Studied by the MCA Test*

| Case | Group | Tissue tested | Histology Deviation from normal: Structure | Histology Deviation from normal: Cellular pleomorphism | Spread | Agglutination | Remarks, etc. |
|---|---|---|---|---|---|---|---|
| 1 | A | Bladder primary (biopsy) | + | + | Submucosa only | +++ | Same tumour re-examined at six weeks interval. |
| 2 | A | Bladder primary (cystectomy) | + | + | Submucosa only | +++ | Normal mucosa also tested. |
| 3 | $A_1$ | Bladder primary (biopsy) | ++ | ++ | Muscle + Lymphatics — | — | — |
| 4 | A | Pleural metastases, fluid aspirate | +++ | + | Widespread metastases | + | Inhibition < 2-fold. Near-diploid tumour. |
| 5 | A | Bladder primary (cystectomy) | + | + | Submucosa only | ++ | — |
| 6 | A | Inguinal node secondary from bladder | ++ | ++ | ? Isolated metastasis 2 yrs. after removal of primary | +++ | — |
| 7 | $A_1$ | Bladder primary (biopsy) | +++ | ++ | Muscle + Lymphatics — | + | — |
| 8 | $A_1$ | Bladder primary (biopsy) | + | + | Submucosa only | ++ | — |
| 9 | A | Bladder primary (cystectomy) | + | + | Submucosa only | +++ | Inhibition 4-fold Normal mucosa also tested. |
| 10 | $A_1$ | Bladder primary (biopsy) | +++ | +++ | Muscle + Lymphatics — | +++ | — |
| 11 | $A_1$ | Bladder primary (biopsy) | + | + | Submucosa only | ++ | Normal mucosa also tested. |
| 12 | A | Carcinoma of renal pelvis (nephrectomy) | + | + | Very early infiltration of renal medulla | +++ | Normal mucosa also tested. |
| 13 | A | Bladder primary (biopsy) | +++ | +++ | Muscle + Lymphatics — | +++ | — |
| 14 | $A_2$ | Bladder primary (biopsy and partial cystectomy) | + | + | Submucosa only | +(+) 50% | Inhibition c. 2-fold. Biopsy and cystectomy specimens gave identical results. |
| 15 | $A_1$ | Bladder primary (biopsy) | + | + | Muscle + Lymphatics — | — | — |
| 16 | A | Bladder primary (autopsy) | +++ | +++ | Wall of bladder, pelvic nodes | ++ | — |

TABLE I—*cont.*

| Case | Group | Tissue tested | Histology Deviation from normal: Structure | Histology Deviation from normal: Cellular pleomorphism | Spread | Agglutination | Remarks, etc. |
|---|---|---|---|---|---|---|---|
| 17 | $A_1$ | Bladder primary (cystectomy) | + | + | Muscle + Lymphatics — | + | — |
| 18 | A | Pelvic metastasis from bladder (autopsy) | +++ | +++ | Widespread in pelvis | — | Inhibition *nil.* Normal mucosa also tested. See Table II. |
| 19 | $A_1$ | Bladder primary (biopsy) | +++ | +++ | Lymphatics + Muscle — | ++ | — |
| 20 | $A_1$ | Bladder primary (biopsy) | + | + | Submucosa only | +++ | — |
| 21 | B | Bladder primary (cystectomy) | +++ | +++ | Muscle + Lymphatics + | — | Normal mucosa also tested. |
| 22 | B | Hepatic metastasis from bladder (autopsy) | +++ | +++ | Widespread in pelvis and liver | — | Inhibition *nil.* |
| 23 | B | Bladder primary (cystectomy) | ++ | ++ | Muscle + Lymphatics — | ++ | — |
| 24 | $A_1B$ | Bladder primary (biopsy) | + | + | Submucosa only | A++++ B+++ | — |
| 25 | $A_1B$ | Bladder primary (biopsy) | ++ | ++ | Muscle + Lymphatics + | A+(+) B+++ | — |
| Controls— A(12), B(6), AB(2) | | Epithelium from renal pelvis, ureters and bladder | — | — | — | +++ | Inhibition tests: A(4), B(2) c. 4-fold. |

*Key to Table I*

*Histology*

| Structure: | | Cytology: | |
|---|---|---|---|
| Well-differentiated | + | Isomorphic | + |
| Poorly-differentiated | ++ | Slightly pleomorphic | ++ |
| Anaplastic | +++ | Markedly pleomorphic | +++ |

*Agglutination*

| | |
|---|---|
| 90–100% | +++ |
| 50–90% | ++ |
| 10–50% | + |
| 0–10% | — |

Whenever possible normal cells from patients with tumours have been tested along with the tumour cells but for a variety of reasons this could not often be done. In operation specimens, for example, it may be difficult to obtain enough normal cells without stromal contaminants or without a proportion of malignant cells which may be infiltrating the submucosa.

In fact in only six cases could a valid comparison be made. From all of these the normal cells gave a +++ or 90–100 per cent reaction whereas in three of the six (Cases 11, 18 and 22) the tumour cell agglutination was reduced or absent.

Inhibition tests with normal cells again give uniform and consistent results. Thus with a cell concentration of 10,000/c. mm. to which is added an equal volume of homologous serum in dilutions up to 1/128 there is normally an 8-fold inhibition. Non-specific adsorption, however, occurs to account for an inhibiting effect of slightly under 2-fold so that the net specific adsorption is in the region of 4-fold (Table II). This has been demonstrated in six cases, four of group A and two of group B.

TABLE II.—*Correlation of MCA Test and Antibody Adsorption test. Negative Result in Latter Test Indicates Adsorption.*

Autopsy (4 hours post mortem) samples of normal ureteric epithelium and secondary deposit of anaplastic bladder carcinoma in pelvis (Case 18).
Blood Group A.
Ureteric suspension : 9600 cells/c. mm.
Tumour suspension : c. 9000 cells/c. mm. Accurate counting impossible.

Agglutination test

| | Ureter | | Tumour | |
|---|---|---|---|---|
| | A cells. | B cells. | A cells. | B cells. |
| Anti-A | +++(100%) | — | —(2%) | — |
| Anti-B | — | — | — | — |

Antibody adsorption test

| Serum dilution | Anti-A Control | Anti-A Ureter | Anti-A Tumour | Anti-B Control | Anti-B Ureter | Anti-B Tumour |
|---|---|---|---|---|---|---|
| Neat | +++ | ++ | +++ | +++ | +++ | +++ |
| 1/2 | +++ | w | +++ | +++ | +++ | +++ |
| 1/4 | +++ | — | ++ | +++ | ++ | ++ |
| 1/8 | ++ | — | + | +++ | ++ | ++ |
| 1/16 | + | .. | — | ++ | w | + |
| 1/32 | — | .. | — | w | — | — |
| 1/64 | — | .. | .. | — | — | — |

In five tumour suspensions subjected to inhibition tests it appeared, not altogether expectedly, that loss of inhibiting activity ran parallel to loss of agglutinability.

Histologically an attempt was made to divide the tumours into three grades of malignancy on two separate criteria, one of structural deviation from the normal and one of cellular irregularity or pleomorphism. With two exceptions these were found to give the same grading. In one case (Case 4), where cells were obtained by aspiration of pleural fluid, a chromosome analysis was performed using the Feulgen squash technique of Ford and Hamerton (1956). Thirty cells counted to an accuracy of $\pm$ 3 contained 40–50 chromosomes and some of these were more definitely near the middle of this range. In other words there was no polyploidy, as was already suggested by the uniformity of the non-mitotic cell nuclei.

In using the agglutination technique the degree of agglutination was at first indicated by a simple system based on numbers of + signs. Subsequently counts of agglutinated and unagglutinated cells have been performed where possible for the sake of greater precision. Negative and strongly positive (+++ or $>$ 90 per cent) results can be taken as reasonably accurate. Intermediate results have shown some variability ($\pm$ 20 per cent) although often there was surprising consistency, e.g. Case 14 where both biopsy and subsequent cystectomy specimens showed almost exactly 50 per cent agglutination.

Agglutination of individual cells does not appear to be an all-or-none phenomenon. Weak positive reactions usually include some completely agglutinated cells but also many cells with a reduced complement of adherent erythrocytes and a correspondingly large area of free surface.

In only one of the negative results has there been complete absence of agglutination. In the other four occasional agglutinated cells, about 1–2 per cent, were noted. It would be easy to ascribe these to artefact were it not that they do not occur in any of the control tubes. Inclusion of a few normal cells is unlikely as great care was taken to sample the most central part of the primary tumour surface. Furthermore they were present in suspensions from metastases.

### DISCUSSION

An analysis of the results as presented in Table I reveals an approximate inverse relationship between the degree of agglutination and the malignancy of the tumour as judged both by microscopy and by the extent of infiltration or metastasis (Table III).

TABLE III.—*Relation of MCA Test to Structure and Spread of Bladder Carcinomata*

| | | Agglutination | | |
|---|---|---|---|---|
| | | +++ | ++ and + | — |
| Spread | Submucosa only | 5 | 4 | 0 |
| | Lymphatics and/or bladder musculature | 3½ | 4½ | 3 |
| | Metastases | 1 | 2 | 2 |
| Histology | Well-differentiated | 6 | 5 | 1 |
| | Poorly-differentiated | 1½ | 3½ | 1 |
| | Anaplastic | 2 | 2 | 3 |

If anything the correlation with tumour-spread is the closer of the two. Thus no tumour limited to the submucosa gave a negative agglutination reaction and only one metastasis gave a strongly positive result. This was a deposit in an inguinal lymph node occurring some two years after removal of the primary vesical carcinoma, there being no other evidence of recurrence. On the other hand one well-differentiated tumour was totally unagglutinable while two anaplastic pleomorphic growths retained normal reactions.

It would seem from the inhibition tests that the negative results were due to inability of the malignant cells to adsorb antibody rather than to failure at a later stage of the agglutination procedure, e.g. inhibition of adsorbed antibody or detachment of loosely bound antibody during washing. It remains to explain this phenomenon.

It is possible but unlikely that there is actual loss of the genes responsible for A and B antigens due to some chromosomal rearrangement in the tumour cells. In favour of this explanation is the behaviour of one of the AB tumours in which a definite distinction was apparent between the A and B reactions. On the other hand two of the pleomorphic tumours, which might be expected to give rise to chromosome-deficient cells, gave normal reactions. Possibly they arose in subjects homozygous for the A gene. A stronger argument against the hypothesis of gene-loss lies in the gradation of reduced agglutinability not so much in cell populations as in individual cells. Genic loss should produce an all-or-none effect which, as has been noted, does not occur.

Alternatively and more probably there occurs some general change in the surface of the cell which masks the A and B antigens. In support of this view one may quote a variety of related phenomena. Irregularity of the surface as viewed by electron microscopy (Coman and Anderson, 1955) a deficiency in the capacity to bind calcium (Dunham, Nichols and Brunschwig, 1946 ; de Long, Coman, and Zeidman, 1950), decreased adherence (McCutcheon, Coman and, Moore, 1948), absence of contact inhibition (Abercrombie and Heaysman, 1953, 1954) and accelerated electrophoretic mobility (Ambrose, James and Lowick, 1956) have all been demonstrated. However the fundamental change underlying these has not yet been elucidated, and it remains to be shown whether any or all of them run parallel to the apparent changes in antigen-content.

The observations here presented may also be relevant to the problem of antibody-resistance in tumours. It seems probable that antigens are not entirely lost by tumour cells since they remain demonstrable to a greater or lesser degree in a proportion. Many cells on the other hand might be able to resist the effects of antibody owing to reduced adsorption at their surface.

On a more practical plane it may be that the method described will give useful information relating to the malignancy of the tumour and the probabliity of infiltration. A drawback here is the immunity of group O cells to antibodies that are easily available. Eel serum anti-H, for example, is ineffective in the test.

### SUMMARY

The epithelium of the human urinary tract contains A and B antigens which are easily demonstrable by the MCA test.

Tumours arising from this epithelium may fail to exhibit these antigens. The implications of this are discussed.

I am indebted to Mr. D. M. Wallace for providing material for this study and to Mrs. A. de Villiers for technical assistance.

### REFERENCES

Abercrombie, M. and Heaysman, J. E. M.—(1953) *Exp. Cell. Res.*, **5**, 111.—(1954) *Ibid.*, **5**, 293.
Ambrose, E. J., James, A. M. and Lowick, J. H. B.—(1956) *Nature*, **177**, 576.
Coman, D. R. and Anderson, T. F.—(1955) *Cancer Res.*, **15**, 541.
Coombs, R. R. A., Bedford, D. and Rouillard, L. M.—(1956) *Lancet*, i, 461
Dunham, L. T., Nichols, S. and Brunschwig, A.—(1946) *Cancer Res.*, **6**, 233.
Ford, C. E. and Hamerton, J. L.—(1956) *Stain Tech.*, **31**, 247.
Green, H. N.—(1954) *Brit. med. J.*, ii, 1374.
de Long, R. P., Coman, D. R. and Zeidman, I.—(1950) *Cancer*, **3**, 718.
McCutcheon, M., Coman, D. R. and Moore, F. B.—(1948) *Ibid.*, **1**, 460.
Weiler, E.—(1956) T. *Naturforsch.*, **11B**, 31—(1956) *Brit. J. Cancer*, **10**, 553, 560.